# Public Snap Map Activity as an Opportunistic Urban Sensor: Spatial Concentration, Temporal Rhythms, and Measurement Requirements Across Three Saudi Cities

**DOI:** 10.3390/s26217018

**Published:** 2026-11-06

**Authors:** Mohammed Almukaynizi, Abdulaziz Alajaji

**Affiliations:** Department of Information Systems, College of Computer and Information Sciences, King Saud University, Riyadh 11543, Saudi Arabia; malmukaynizi@ksu.edu.sa

**Keywords:** social sensing, opportunistic sensing, Snap Map, urban activity sensing, sensor characterization, spatial concentration, Gini coefficient, ephemeral media, volunteered geographic information, Saudi Arabia

## Abstract

Public Snap Map activity, a short-lived stream of stories pinned to places, can serve as an opportunistic urban sensor, and this study measures what it reports and what it requires to report reliably. From more than 1.18 million public snaps collected on a 1 km grid across three contrasting Saudi cities in 2024, activity is highly concentrated, to degrees that differ by city. Over the full windows, Makkah leads on every measure (Gini 0.71 [0.68, 0.73]), with one Grand Mosque tile holding 2.7% of its snaps, and its lead survives a spatial-block bootstrap. Riyadh (0.59 [0.57, 0.60]) and Dammam (0.56 [0.54, 0.59]) are close in degree and differ in shape: Dammam concentrates in a strong head of busy tiles, Riyadh in a long active tail. Over the week that all three windows share, Makkah does not lead, because its estimate rises with accumulated observation. Two operating requirements follow. Concentration estimates stabilized after 23 to 27 days in the two campaigns observed long enough to confirm it and after 12 to 14 days once collection start-up had passed. Under binomial thinning with item-independent visibility, the Gini is biased downward, Makkah’s lead survives at 5% retention, and the Riyadh–Dammam ordering inverts at 10%.

## 1. Introduction

Cities leave measurable traces in the geographic content their inhabitants volunteer, and the citizens-as-sensors paradigm treats those traces as readings from a distributed instrument [1]. A long line of urban-computing work has built on this premise: individually noisy posts, aggregated over space and time, resolve into stable pictures of where a city is busy and when [2], and the recent growth of spatially explicit artificial intelligence has supplied formal models and benchmarks for exactly this kind of signal [3,4]. Such a platform is a sensor in a specific and demanding sense. It is never calibrated, its sampling is opportunistic rather than designed, and the analyst controls neither where it observes nor how much of what it observes is reported. Using one responsibly therefore requires knowing its operating characteristics: how long it must observe before a measurement settles, how much of its output can be lost before a conclusion changes, and which comparisons survive that loss. These characteristics have been established for the persistent, text-rich sources that shaped the field’s methods—Twitter, Flickr, and Foursquare [5,6,7,8]—where a sampled stream can be checked against a re-queryable archive: Morstatter et al. compared Twitter’s sample against its firehose, and Pfeffer et al. showed that the sample can itself be manipulated [9,10]. Two recent reviews confirm that the field remains anchored on these established, queryable streams [11,12]. For ephemeral visual platforms these characteristics are unknown, and such a check is not possible.

Snapchat’s Snap Map is worth measuring because it is one of few public sources that record where and when people post, at fine spatial resolution and with little delay. It has been used to study crowd behavior at a mass-gathering site [13] and to follow unfolding events [14]. With its operating properties measured, it can complement surveys and footfall counters in urban-vitality work [15] as a low-latency indicator of where and when platform activity concentrates. Snapchat’s potential advertising reach covers 91.8% of the Saudi population aged 13 and above [16], which makes Saudi cities a natural test.

Snap Map is a distinctive and under-measured member of that class of sensors. It exposes a continuously refreshed, public stream of short stories pinned to precise coordinates. Its content is hyper-local, visual, and *ephemeral*: expired or deleted stories vanish from the public map. This ephemerality is the medium’s defining design: a channel for sharing small, in-the-moment fragments of daily life rather than a durable archive [17]. It sets Snap Map apart from the persistent, identity-anchored sources that shaped the field’s methods, such as geolocated tweets, check-ins, and geotagged photographs, whose payload can be archived and re-queried and whose records are tied to an account [18,19,20]. Ephemerality is what makes it an attractive but awkward instrument. Because expired content leaves no archive, the stream cannot be re-queried after the fact: a measurement not taken while the window was open cannot be recovered, which makes knowing the sensor’s requirements in advance a practical necessity. Direct measurements of Snap Map are scarce. The closest quantitative study examined three U.S. cities [21]. Whether its findings generalize beyond the U.S. is still an open question, especially for a Gulf setting whose polycentric, car-oriented form and Friday–Saturday weekend differ structurally from the U.S. cases.

We measure what such a sensor reports with a well-tested toolkit for quantifying how unevenly activity concentrates in space. The Gini coefficient [22] summarizes in one number how unevenly snaps are spread across map tiles (0 when every tile holds the same number, approaching 1 as snaps gather into a single tile; Equation (1)), and it is derived from the Lorenz curve [23]. These sit alongside the observation, established on persistent platforms, that spatial and temporal signatures of user-generated activity differ systematically across kinds of places [24,25]. The only quantitative Snap Map measurements come from broadly similar U.S. cities and report one pooled top-share figure—about a quarter of snaps from the most active 1% of tiles—rather than a per-city, whole-distribution scalar with stated uncertainty [21]. The Gulf-region Snap Map work we are aware of examines one site, the Grand Mosque, with no cross-city concentration analysis [13]. None of them reports how long the stream must be observed, or how much of it must be captured, for such a measurement to hold.

**Research gap.** To our knowledge, no prior work characterizes public Snap Map activity as a measurement instrument: no study reports its spatial concentration across several cities as a comparable scalar with stated uncertainty, and none establishes the observation length or capture fraction its readings require. This article closes that gap. We analyze more than 1.18 M public snaps across three cities chosen for their contrasting urban roles—administrative Riyadh, religious Makkah, and industrial/coastal Dammam—with tile and spatial-block bootstrap confidence intervals (CIs) and sensitivity analyses. Makkah is the most spatially concentrated city by every measure (Gini 0.71); Riyadh and Dammam are close in degree and concentrate in different ways. Two operating requirements follow, and they bound what a deployment of this sensor can claim.

**Contributions.** The study contributes a comparable multi-city measurement of this sensor with stated uncertainty, as well as a count-based procedure, items (iv) and (v), demonstrated here on Snap Map and set out in general form in Section 4.3, that shows how its conclusions depend on the observation length, the capture fraction, and the definition of the active area. Specifically, the contributions are as follows: (i) A large-scale, multi-city measurement of public Snap Map activity (>1.18 M snaps; Riyadh, Makkah, Dammam) under a common collection methodology. (ii) Quantification of spatial concentration (Gini, Lorenz, top-share) with tile and spatial-block bootstrap uncertainty, showing that concentration is two-dimensional: Makkah leads on both the head and the whole-distribution measures, while Dammam concentrates in a strong head of busy tiles and Riyadh in a long active tail. (iii) Temporal profiles (hour-of-day; day-of-week normalized for weekday occurrence) that separate the three cities, with a matched-window sensitivity analysis across the seven days all three collections share, and a matched-observation-length comparison that removes the confound of unequal windows. (iv) Two measurement properties of this sensor, as observed here: *integration time*, namely the 23 to 27 days over which a concentration estimate stabilized in the two campaigns whose windows outlasted their own estimates (12 to 14 once collection start-up had passed), with shorter windows understating concentration, except where activity had already accumulated; a *sampling sufficiency* curve obtained by binomial thinning, which under approximately item-independent partial visibility shows that the Gini is biased downward yet leaves Makkah’s lead intact at 5% retention, while the Riyadh–Dammam ordering inverts at and below 10% retention, with a clustered variant for loss by place and time. (v) A closed-form expression for how much a concentration comparison depends on where the active support is drawn, converting an untestable modeling choice into a reportable margin. (vi) Public archiving of the tile-level aggregates for all three cities and of the analysis code.

## 2. Related Work

We group prior work into five threads: the volunteered geographic information (VGI) and geosocial-sensing paradigm, inference of urban function from geosocial activity, temporal rhythms and urban vitality, the measurement of spatial concentration and VGI bias, and the small body of work on Snap Map itself. We then position the present study in Table 1.

### 2.1. VGI and Geosocial Sensing

The notion of “citizens as sensors” [1] established volunteered geographic information as a complement to authoritative mapping, treating the geotagged output of ordinary users as a continuously updated public observation stream. This paradigm has been applied across data sources and tasks. Crandall et al. [6] mapped points of interest worldwide from the spatial clustering of geotagged Flickr photos. Noulas et al. [7] characterized recurrent human activity from Foursquare check-ins. Cranshaw et al. [8] recovered functional “livehoods” from the co-visitation structure of social check-ins, and Sakaki et al. [5] detected earthquakes in near real time from Twitter. These studies sit within a broader tradition of urban computing that turns heterogeneous human traces into a city-scale signal [2] and alongside mobility research showing that individual movement is highly regular [26]. Recent surveys update the paradigm [11,12]. A common feature of these sources is that content is *persistent* and largely *text-centric*: a tweet, check-in, or captioned photo remains queryable long after it is posted. Snap Map differs on both axes. Its content is visual and, more importantly, *ephemeral*, so the public record at any instant is only what has not yet expired or been deleted. This changes what can be measured, which is a near-live cross-section rather than an accumulating archive. It also changes how the measurement must be done—through continuous polling rather than one-time retrieval.

This framing has an engineering counterpart. Mobile phone sensing and mobile crowdsensing turn participant-carried devices into a distributed instrument, and the surveys of that field are organized around the questions asked here: How much participation suffices? And how good is the information it yields? [27,28,29]. The difference is control. Those systems own the sampling schedule, can recruit or re-task participants, and can re-query a device that returned nothing; a public ephemeral stream offers none of the three possibilities, so its operating characteristics must be measured after the fact, from the record it leaves, rather than designed in.

### 2.2. Inferring Urban Function from Geosocial Activity

A substantial body of literature infers urban structure and land use from geosocial activity. Frias-Martinez and Frias-Martinez [24] applied spectral clustering to geolocated Twitter activity and recovered interpretable land-use classes (residential, business, leisure, nightlife) from temporal activity signatures alone. Li et al. [30] showed that the spatial, temporal, and socioeconomic patterns of Twitter and Flickr use vary systematically across places, and Steiger et al. [31] cataloged, in a systematic review, the breadth of spatiotemporal analyses built on geosocial streams. The current frontier is urban functional zone (UFZ) identification. Mawuenyegah et al. [32] mined spatiotemporal geosocial patterns for UFZs. Luo et al. [33] and Xu et al. [34] classified zones from point-of-interest (POI) density with machine learning and graph autoencoders. Liu et al. [35] fused mobile-signaling and POI data with deep learning, and Zhi et al. [36] mapped green-space functional zones from remote sensing combined with crowdsourced data. This frontier rests on an older foundation that tied activity to place function, from discovering regions of distinct function through human mobility and points of interest [37] to inferring land use from the temporal signature of mobile-phone activity [38]. This body of work shapes our design in two connected ways. *Temporal activity profiles are themselves diagnostic of function*, so the same logic that lets a daily rhythm separate a nightlife district from an office district should let a city-level rhythm separate a religious city from an administrative one; yet this modern, method-heavy UFZ literature rests almost entirely on Western or Chinese cities and never touches an ephemeral Snap Map stream or a Gulf urban form. The present study addresses this gap with a transparent measurement.

### 2.3. Temporal Rhythms and Urban Vitality

A parallel thread treats the temporal profile of activity as a first-class signal. Du et al. [25] linked the daily rhythm of urban space usage to urban function and human mobility. Knoblauch et al. [39] provided a long-term validation of inner-urban mobility metrics derived from Twitter/X against mobile-phone ground truth, cautioning against short-term inference. Karasov and Järv [40] mapped activity locations from social media enriched with time-use and satellite data. A related body of urban-vitality literature quantifies how alive a place is from big geo-data. Kim [15] measured vitality from big data and the Internet of Things. Jiang et al. [41] related urban morphology to vitality, and Liu et al. [42] combined built-environment and perception dimensions. These studies establish that diurnal and weekly activity profiles are interpretable and tied to function, but they draw almost entirely on persistent platforms or device traces, study individual cities or homogeneous city sets, and do not pair the temporal signal with a quantified spatial-concentration measure in a single comparative frame. The weekend cycle they assume (Saturday–Sunday) also does not transfer to a Gulf calendar. The official weekend in Saudi Arabia has been Friday–Saturday since a royal decree in June 2013, when it moved from the earlier Thursday–Friday. This article exploits that contrast.

### 2.4. Measuring Spatial Concentration and VGI Bias

To quantify how unevenly activity is distributed across space we adopt the Lorenz curve and the Gini coefficient [22], the standard summary of distributional inequality, computed here over per-tile snap counts (Section 4). The Gini coefficient reduces an entire concentration profile to a single comparable scalar in [0,1], which is what allows a clean cross-city ordering; we report the full Lorenz and rank–size curves alongside it so the scalar is not read in isolation. Recent urban-data work applies related concentration and segregation measures to other targets. Li et al. [43] argued for a big-data paradigm of segregation measurement. Gao et al. [44] unpacked socio-spatial inequalities in mobility via urban scaling, and Poorthuis et al. [45] released a nationwide activity-space dataset from geotagged social media. The bias literature supplies the necessary caveat: Hecht and Stephens [46] documented urban biases in VGI, and Fan et al. [47] showed that social-media attention concentrates in populous areas during disasters. The closest antecedent to the present measurement is Indaco and Manovich [48], who applied the Gini coefficient to 7.4 million geotagged Instagram images in Manhattan, across census tracts and across a 24 h cycle. That literature applies concentration metrics to mobility, segregation, disaster attention and—in that one case—platform activity density, but always within a single city rather than compared across functionally distinct ones; none of it addresses Snap Map, and none asks how long a stream must be observed, or how much of it captured, for such a measure to hold. It also motivates framing our corpus as a measurement of *platform-visible* activity, a lower bound rather than a count of the underlying population. We return to this point in Section 7.

### 2.5. Snap Map Specifically

Direct measurements of Snap Map are scarce and concentrated in a few groups. The closest quantitative study is that of Juhász and Hochmair [21], who analyzed Snap Map’s spatial and temporal dynamics across three U.S. cities and reported strong concentration of activity in dense urban cores, pooling the three into a single figure of about a quarter of snaps in the most active 1% of tiles. The same authors subsequently compared Snapchat against Twitter and Flickr in Florida and found that its spatial and temporal contribution patterns differ systematically from those of the older, persistent platforms [49]. Outside this quantitative line, the platform has been studied mainly qualitatively—for example, as a medium for digital placemaking [50]. For the Gulf framing, the most relevant work is by Alghamdi et al. [13], who used Snap Map to study crowd behavior at the Grand Holy Mosque in Makkah. Their study shows that Snap Map carries a usable, place-specific signal in a Saudi setting, though it is a preliminary single-site study without concentration metrics. This thin and U.S.-centric literature leaves three questions open that motivate our study. Does the concentration pattern hold *outside the U.S.*? Does it differ between cities less alike than three large U.S. metros? And what does a whole-distribution measure with stated uncertainty show that a single pooled top-share figure cannot? The present article addresses these questions by adding two further Saudi cities with different urban roles and introducing concentration metrics with stated uncertainty, in a Gulf setting absent from prior studies. Table 1 situates this work against the most comparable studies.

## 3. Data and Collection

This study draws on a public corpus of Snap Map activity, retrieved through the platform’s public map interface. This article uses the three-city public-snap catalog: it analyzes metadata only and computes no feature from media of any kind.

### 3.1. The Snap Map Public Stream

Snap Map is the map view within Snapchat. Besides sending content privately to friends, a user can submit a photo or short video to the public map from the app’s send screen; submissions pass automated review, and accepted ones may be shown at the place they were posted to anyone browsing that area [51]. Selfies, Memories, and camera-roll content do not appear on the map, and a user may submit without associating the snap with their name [51,52]. The platform fixes no display period: some submissions stay viewable for a day or two and others much longer [51]. The map surfaces a changing selection of recent stories at each place, so a story not captured while surfaced cannot be retrieved later through the same interface. The public stream, as collected here through the public web interface without logging in, carried no username or profile. In a test during the Riyadh deployment, posted videos appeared on the map within 12 min [53].

The collection platform and its Riyadh deployment are described in a companion paper [53], which also reports descriptive Riyadh results from the same window: snap counts, daily volume, a heatmap, the share held by the busiest tenth of tiles, and the saturation test discussed in Section 3.5. It reports no Gini or Lorenz analysis, no other city, and no measurement requirements, which are the contributions of this article.

### 3.2. Collection Windows

The catalog was collected over a different window in each city. It holds 1,209,102 records across three Saudi cities—Riyadh with 534,389, Makkah with 280,803, and Dammam with 393,910. Restricted to each city’s core collection window, it holds 511,918 Riyadh records over the 23 days from **17 September to 9 October 2024**, and 278,044 Makkah and 391,103 Dammam records over the 32 days from **3 October to 3 November 2024**. The windows are largely disjoint but not entirely: all three overlap on seven days, **3–9 October 2024**, a span that contains each day of the week exactly once and no national holiday. Outside that week the cities were observed at different calendar times, so no claim made here compares the three cities at matched calendar time unless it says so explicitly. Riyadh records were collected inside the bounding box latitude 24.4–25.0, longitude 46.4–47.0. Because each window is bounded, the corpus supports within-window spatial and temporal structure rather than long-term trends. Aggregate activity counts for the Riyadh portion of this collection are archived separately [54].

Table 2 gives the records remaining at each step, from the catalog to the analyzed corpus. Two further Riyadh figures circulate near this work, and what separates them is a day-boundary convention rather than a different corpus. This program dates every record in Riyadh local time (UTC+3), under which the 17 September–9 October window holds 511,918 records over 23 days; removing 29 September, an incomplete day carrying 480, leaves the 511,438 analyzed here. The companion paper [53] dates the same window in Coordinated Universal Time (UTC), under which it holds 515,364, or 514,543 once that day is removed. It also counts 2271 distinct stored coordinates rather than 2219 query-point labels (Section 5.3). Only the Table 2 figures enter the results reported here.

### 3.3. Collection Pipeline

We collected public snaps with a grid-based pipeline that polls the Snap Map tile service; the platform that implements this pipeline, including its application programming interface (API) validation and collection management, is documented for the Riyadh deployment in the companion paper [53]. Makkah and Dammam were collected with the same pipeline in a later window. For each city we lay down a regular lattice of query points spaced at ∼1 km. We chose this spacing as a deliberate design parameter: it is fine enough to separate adjacent neighborhoods yet coarse enough that the campaign covers a full metropolitan area within the service’s request limits, and, as the coverage analysis in Section 3.5 shows, adjacent points at this spacing return heavily overlapping stories, so a denser lattice would add cost without adding coverage. We retain only points that fall inside the city’s administrative boundary. Boundary membership is decided by a point-in-polygon test against the public district-boundary dataset of [55], so a query point is kept if it lies within any district polygon of the target city. Points exactly on a shared boundary are assigned to a single district by the first containing polygon in dataset order, so a snap is never double-counted across districts. Each retained point is polled repeatedly over the collection window. The fields this study uses are, for every returned story, the location of the query point that first retrieved it (not the poster’s precise location), the server timestamp, the media type, and the containing district (Table 3). No user identifier is collected.

### 3.4. Deduplication and Filtering

Because adjacent query points and successive polls return overlapping stories, the raw polling stream contains many repeated observations of the same snap. Deduplication happens at collection time: each returned story carries a platform-assigned media identifier, and the pipeline stores a record only for identifiers not already in the database, so each public story enters the catalog once. The archived catalog is accordingly already unique on the media identifier; the analysis stage re-applies the check defensively, and, as expected, it removes zero records in all three cities. We then aggregate the snaps to 1 km tiles indexed by their query point. Two selection rules produce the analyzed corpus. The first restricts each city to its stated core window; the catalog also holds records from an earlier collection episode and from boundary days outside the windows analyzed here. The second drops any calendar day whose snap count is below 1% of the busiest day in that city’s window, since a fractionally observed day would bias temporal and volume statistics. This rule leaves Makkah’s and Dammam’s 32 window days intact and removes a single low-coverage day inside Riyadh’s window (29 September, 480 snaps), leaving 22 analyzed days. The collection on that day was interrupted by a scheduler failure and restarted the same evening [53]. The value of 1% is not critical: any threshold from 0.77% to 6.56% of the busiest day removes that day and no other, and Makkah and Dammam keep all 32 days for any threshold up to 16.03%. Finally, all reported per-tile statistics run over *active* tiles, meaning tiles with at least one snap. Empty grid points are an artifact of the lattice rather than an observation, so we exclude them from the distributional metrics; the sensitivity analysis in Section 5.3 probes this choice. Table 2 summarizes the three datasets over their 2024 windows. After windowing and filtering the corpus exceeds 1.18 million public snaps.

### 3.5. Sampling and Coverage Validation

The tile service is undocumented, so the fraction of all posted stories it exposes is unknown. What can be measured is whether this visible fraction is *stable under repeated querying*, which is a prerequisite for treating the measurement as a reliable signal. To check this, we use the scraper’s own per-run logs. For each neighborhood on each repeated pass over the grid, these logs record how many returned items were newly seen and how many were duplicates of already-collected snaps. The probe is a saturation experiment run over a short window (7–10 October 2024) that sits *inside* Riyadh’s longer collection campaign. By the time the probe began, the Riyadh corpus was already partly built, so the probe measures how quickly repeated passes stop adding new media rather than building the corpus from empty. Over the 21 repeated passes in this window the scraper logged 4,799,943 item observations (≈4.8 M). Of these, 4,551,216 (94.8%) were duplicates of snaps already seen and only 248,727 (5.2%) were newly observed media. These ≈250 K newly seen items are not the whole campaign. They are the new media surfaced *during this probe window*, a subset of Riyadh’s full deduplicated corpus of 511,438 snaps (Table 2) that the same pipeline accumulated over the full window. The probe quantifies the *marginal* return of re-querying an already-populated grid. It is not a re-collection of the entire campaign and is not expected to recover the full corpus count. Repeated queries thus overwhelmingly re-observe the same public stories rather than surfacing large hidden pools. Coverage of the platform-visible set saturates, and the deduplicated corpus is stable rather than an arbitrary slice.

The same per-run logs were retained for Makkah and Dammam, covering 10 October to 4 November 2024 (107 runs each), that is, from each collection’s second week onward. Across those runs, 96.9% and 96.5% of returned items were snaps already in the catalog (medians per run 98.0% and 97.4%), against 94.8% in the Riyadh probe, so repeated polling saturates in all three grids. Saturation does not fix the absolute sampling fraction, which the platform does not expose; the results therefore describe *platform-visible* activity (Section 7).

## 4. Methods

For each city we aggregate the deduplicated snaps to 1 km tiles and analyze the resulting per-tile count distribution and the temporal distribution of snaps. All quantities are computed by the archived analysis scripts over the filtered windows.

### 4.1. Spatial Concentration

Let $x_{1}\leq x_{2}\leq \cdots \leq x_{n}$ be the snap counts of the *n* active tiles in a city, sorted in non-decreasing order. The *Lorenz curve* plots the cumulative share of snaps against the cumulative share of tiles; the diagonal corresponds to a perfectly even distribution. We summarize the curve by the *Gini coefficient:*

(1) $$G\;=\;\frac{1}{n}\left(n+1-2\;\frac{\sum_{i=1}^{n}\left(n+1-i\right)\;x_{i}}{\sum_{i=1}^{n}x_{i}}\right),$$

with 0 for a perfectly even spread across tiles, and values near 1 for activity concentrated in a single tile (Equation (1) peaks at (n−1)/n). Read plainly, the Lorenz curve answers what share of snaps the least active fraction of tiles holds, and the Gini is the gap between that curve and perfect evenness. We use the Gini because it summarizes the whole distribution on a bounded scale and was used in the closest prior study of platform activity [48]; top-share measures complement it by describing the head of the distribution. We also report the *top-1% share*, the fraction of all snaps falling in the busiest 1% of tiles, and the busiest single tile. We plot the *rank–size* distribution (snaps per tile versus rank, log–log) so that the head and tail of each city’s distribution are visible alongside the scalar *G*.

### 4.2. Temporal Structure

Temporal structure is summarized by the distribution of snaps over *hour-of-day* (0–23) and *day-of-week* (Mon–Sun) in local time. Riyadh was observed for 22 analyzed days between 17 September and 9 October 2024, and Makkah and Dammam for 32 days between 3 October and 3 November 2024. Only Riyadh’s window contains Saudi National Day. The windows share one week, 3–9 October, which opens the Makkah and Dammam collections and closes Riyadh’s; Section 5.3 uses it and a matched observation length as sensitivity comparisons. Because the windows differ in length, absolute snap volumes are not directly comparable across cities. We therefore express every temporal profile as a *within-city percentage*, normalizing each city’s snaps to sum to 100% over the relevant axis. For the day-of-week axis a further normalization is required: the windows are not whole multiples of seven days, so weekdays occur unequal numbers of times (Makkah’s and Dammam’s 32-day window holds five Thursdays through Sundays but only four Mondays through Wednesdays, and Riyadh’s 22 retained days range from two Sundays to four Tuesdays). A raw share would inflate the weekdays that merely occur more often, so the day-of-week profile is the mean count per *occurrence* of each weekday, renormalized to sum to 100%. This way the *shape* of a city’s rhythm, not its raw volume or its window’s calendar composition, drives the cross-city comparison. For the volume comparisons that do appear, such as busiest-tile counts, we report both the raw figure and a per-day rate (snaps divided by the number of retained collection days). The reader can then separate genuine differences in intensity from differences in window length. Finally, we attach 95% CIs to the concentration statistics with two nonparametric bootstraps of 5000 resamples each under a fixed seed. A bootstrap redraws the tiles many times with replacement and recomputes the statistic each time; the spread of the results shows how much the estimate depends on which tiles happened to be observed, not on how the city was sampled in time. The first bootstrap resamples tiles independently. Because neighboring tiles are correlated, the second resamples square spatial blocks of query points, 5 km on a side (10 km in Supplementary Table S1), on a metric grid aligned to each city’s query lattice. Between-city differences are reported as percentile intervals of the bootstrap distribution of the difference, with the cities resampled independently.

### 4.3. Measurement Requirements

Two properties of the stream as an instrument are characterized by re-analyzing the collected corpus. The *integration time* is obtained by recomputing the concentration statistics over the corpus accumulated from the first day of a city’s window through day *k*, for every *k*, and taking the smallest *k* beyond which the running estimate never again departs from its final value by more than 0.01 in Gini units. Searching from the end of the series rather than the start ensures that a curve that briefly touches the tolerance band and then leaves it is not recorded as settled. The rule is repeated at tolerances of 0.005 and 0.02 and on curves restarted once a collection’s start-up had passed, defined as the first day that opens a run of seven days on each of which the number of active tiles reaches 85% of its median over the window’s final 14 days. Two properties of this definition bound how its output may be read. The reference value is the estimate at the end of the observed window rather than an unobservable long-run value, so the statistic records when a curve stops moving relative to where it happened to stop. And the search is bounded by the window: a settle point at or near the last day is tested against only the one or two observations that follow it, so it is right-censored and reports a lower bound rather than a measurement. A window must therefore be appreciably longer than the integration time it is used to estimate. *Sampling sufficiency* is characterized by binomial thinning, which simulates seeing only part of the stream: each snap is retained independently with probability p∈{0.05,0.10,0.25,0.50,0.75}, the tile distribution is rebuilt from the retained counts over the tiles that remain active, and the statistics are recomputed, with 200 repetitions per rate under a fixed seed. This models item-level visibility independently across snaps. A clustered variant keeps or drops whole tile-days with probability *p* so that visibility depends on place and time together, with the support again recomputed from the retained tiles (Supplementary Table S3). Both procedures describe the behavior of the aggregate estimator; whether individual tiles retain their rank from one day to the next is a separate question about the persistence of the underlying activity.

**The procedure in general form.** These analyses, together with the support identity applied in Section 5.3, form a procedure that requires only event counts per fixed spatial unit and time bin. Its use on other ephemeral geolocated streams that yield such counts is a potential extension, not tested here. (1) *Integration time*: Recompute the concentration statistic on the corpus accumulated through each day, and report the earliest day after which it stays within a tolerance δ of its end-of-window value, at several tolerances and after start-up. (2) *Sampling sufficiency*: Retain events independently, or whole unit-days, with probability *p*; recompute the active support and the statistic over repeated draws; and report the tested rates at which each between-group ordering holds or reverses. (3) *Support margin*: For *n* active units with Gini *G*, adding *Z* zero-count units gives $G^{\prime }=1-\frac{n}{n+Z}\left(1-G\right)$, so the number of added units that would equalize two groups is a reportable margin. The outputs for each comparison are a settling time, a retention-sensitivity curve, and a support margin; the archived notebook [56] computes all three.

All analyses were implemented in Python 3.13.9 (Python Software Foundation, Wilmington, DE, USA) with pandas 2.3.3, NumPy 2.3.5 and Matplotlib 3.10.6 and run deterministically from the stored data tables with fixed random seeds. A generative AI assistant (Claude, Anthropic) was used during the development of the analysis code and for independent numerical cross-checks of the computed statistics; the authors reviewed and verified the code and every reported number, and the assistant was not used for data collection or for drawing inferences.

## 5. Results

### 5.1. Spatial Concentration Across the Three Cities

**Figure 1:**
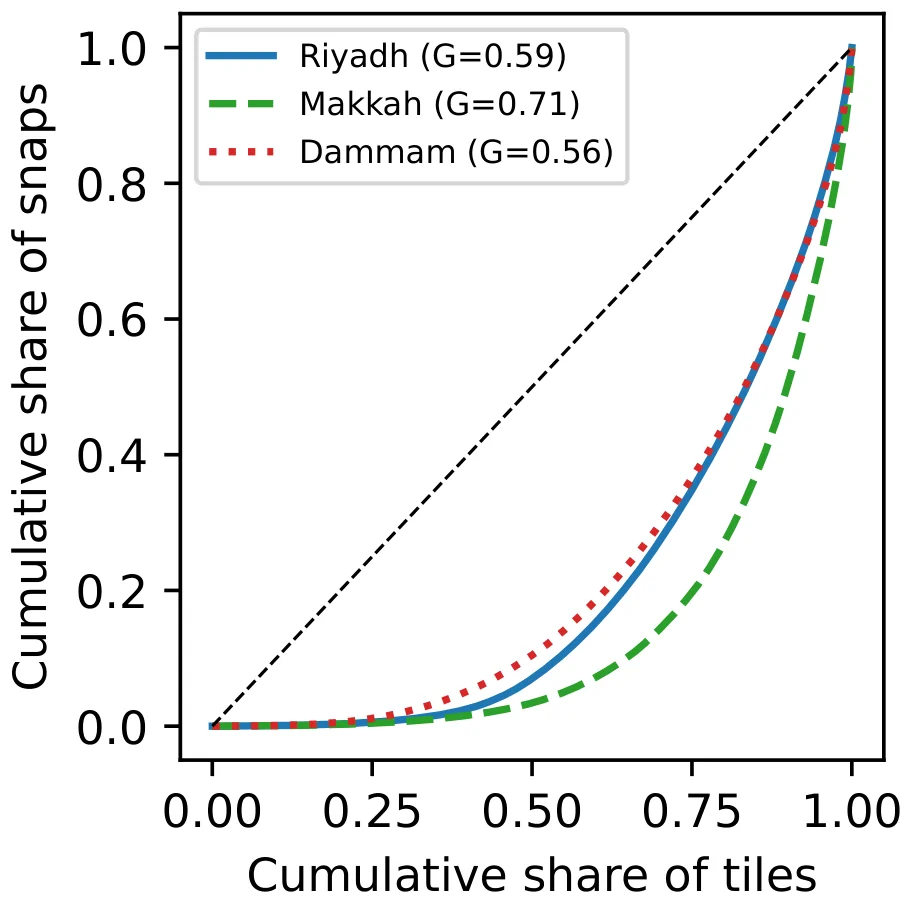
Lorenz curves of snaps per tile. Makkah deviates the most from equality (Gini 0.71); Dammam is the most even (Gini 0.56). The diagonal is perfect equality.

The cities differ in how concentrated their activity is, and the difference is not a single ordering but a two-dimensional pattern (Figure 1, Table 4). *Makkah* leads on every concentration measure: G=0.71 [0.68, 0.73], the top 1% of tiles holding 10.7% of all snaps, and a single 1 km tile at Masjid al-Haram (the Grand Mosque) containing 7379 snaps, 2.7% of the entire city’s corpus. This is 3.5× the raw count of Riyadh’s busiest tile (2087). Because Riyadh’s window is shorter, we also compare per-day rates. The gap remains large but narrows: Makkah’s busiest tile averages ∼230.6 snaps/day against Riyadh’s ∼94.9, a factor of ∼2.4. Makkah’s Gini sits roughly nine bootstrap standard errors above the next city’s, so its lead is not an artifact of tile-sampling noise. It exceeds Riyadh’s by 0.117, with a 95% interval for the difference of [0.092, 0.143] under the tile bootstrap and [0.053, 0.177] with 5 km spatial blocks, so its lead holds under spatial dependence. *Riyadh* (G=0.59 [0.57, 0.60]) and *Dammam* (G=0.56 [0.54, 0.59]) are close in degree: their difference of 0.027 has intervals of [−0.003, 0.054] (tiles) and [−0.047, 0.100] (blocks) and, at matched observation length (Section 5.3), of [0.009, 0.068] and [−0.035, 0.112]. Riyadh’s estimate is the least converged of the three (Section 5.4), and because running estimates approach their final value from below, longer observation of Riyadh would move it away from Dammam rather than toward it. On the head-based measures they reverse, with Dammam’s top-1% share (7.3%) and busiest tile (5573 snaps, 174.2/day) both exceeding Riyadh’s. The two cities concentrate *differently* rather than more or less: Dammam pairs a strong head with by far the highest median tile (298.0 snaps, versus 152.0 for Riyadh and 67.0 for Makkah), with activity gathered into many busy tiles across the Dammam–Khobar–Dhahran area, whereas Riyadh spreads a comparable total over nearly three times as many active tiles (2219 versus 848), a long tail of moderately active locations. Makkah’s low median beneath an extreme maximum reflects a single dominant core.

**Figure 2:**
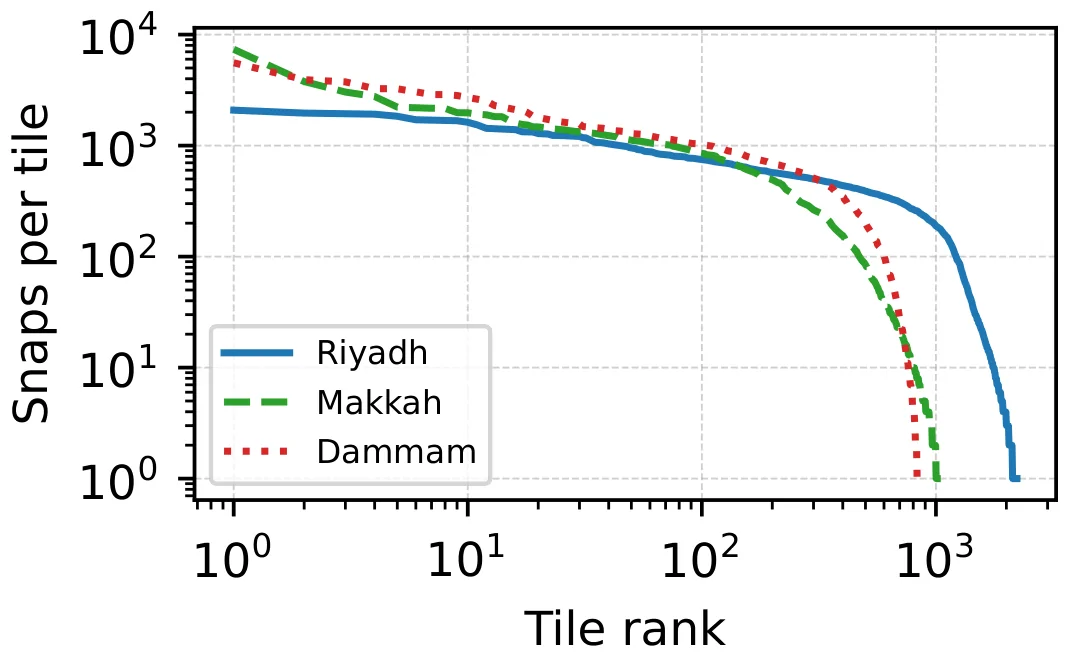
Rank–size distribution of snaps per tile (log–log). Makkah has the steepest head and Dammam the flattest head; Riyadh extends furthest along the rank axis, reflecting its larger count of active tiles.

**Figure 3:**
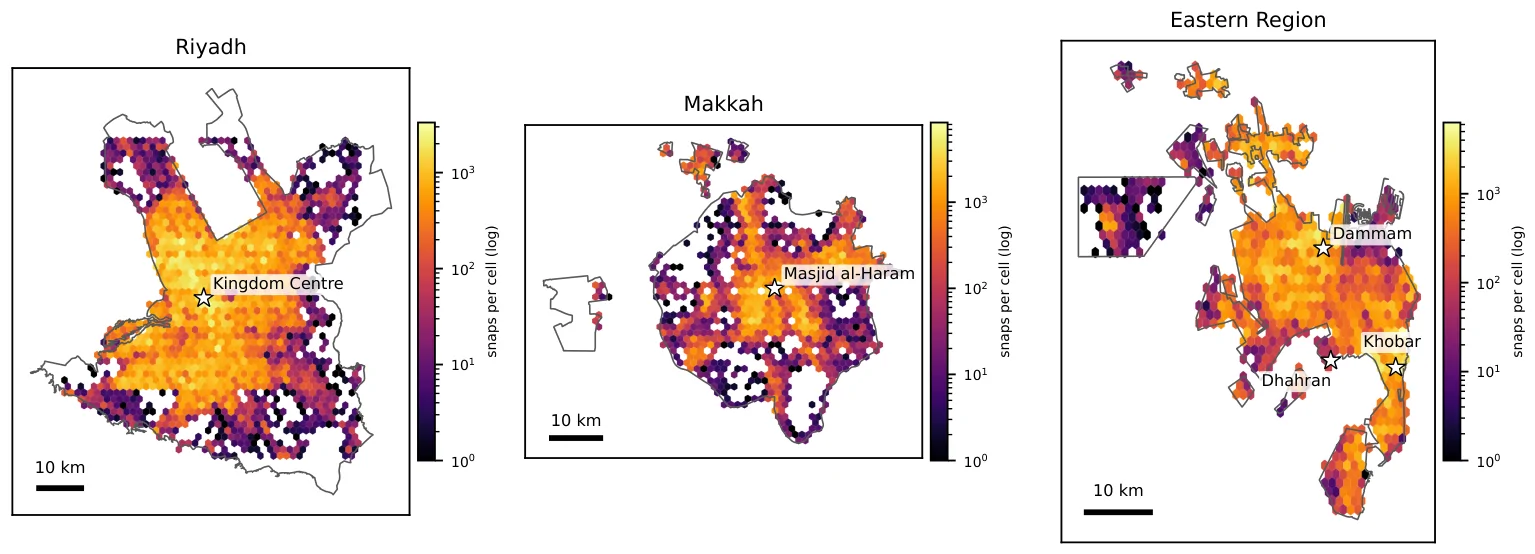
Snap-density heatmaps with a scale bar, the outline of the sampled districts, and landmarks. Each panel uses its *own* per-city log color scale (brighter = higher density *within that city*). The panels are *not* on a shared scale, so brightness is not comparable in absolute terms across cities, and the figure shows within-city relative density rather than cross-city absolute density. The comparison to draw is the *spatial spread* of each city’s activity. Makkah concentrates tightly around the Grand Mosque in a single dominant core; the Eastern Region panel spreads across the Dammam–Khobar–Dhahran coastal area; Riyadh is broadly distributed. This spread ordering, from most to least concentrated, matches the Gini coefficients in Table 4.

The rank–size curves (Figure 2) show the same ordering from the head of the distribution: Makkah has the steepest fall-off into a single dominant tile, whereas Dammam’s head is the flattest, with many tiles of comparable size. Riyadh’s curve extends furthest along the rank axis, reflecting its much larger count of active tiles (2219). The snap-density heatmaps (Figure 3) make this spatial structure visible. Makkah collapses to a single bright core at the Grand Mosque, Dammam spreads across its coastal area, and Riyadh is broadly distributed. This visual ordering matches the Gini coefficients.

### 5.2. Temporal Rhythms Across the Three Cities

**Figure 4:**
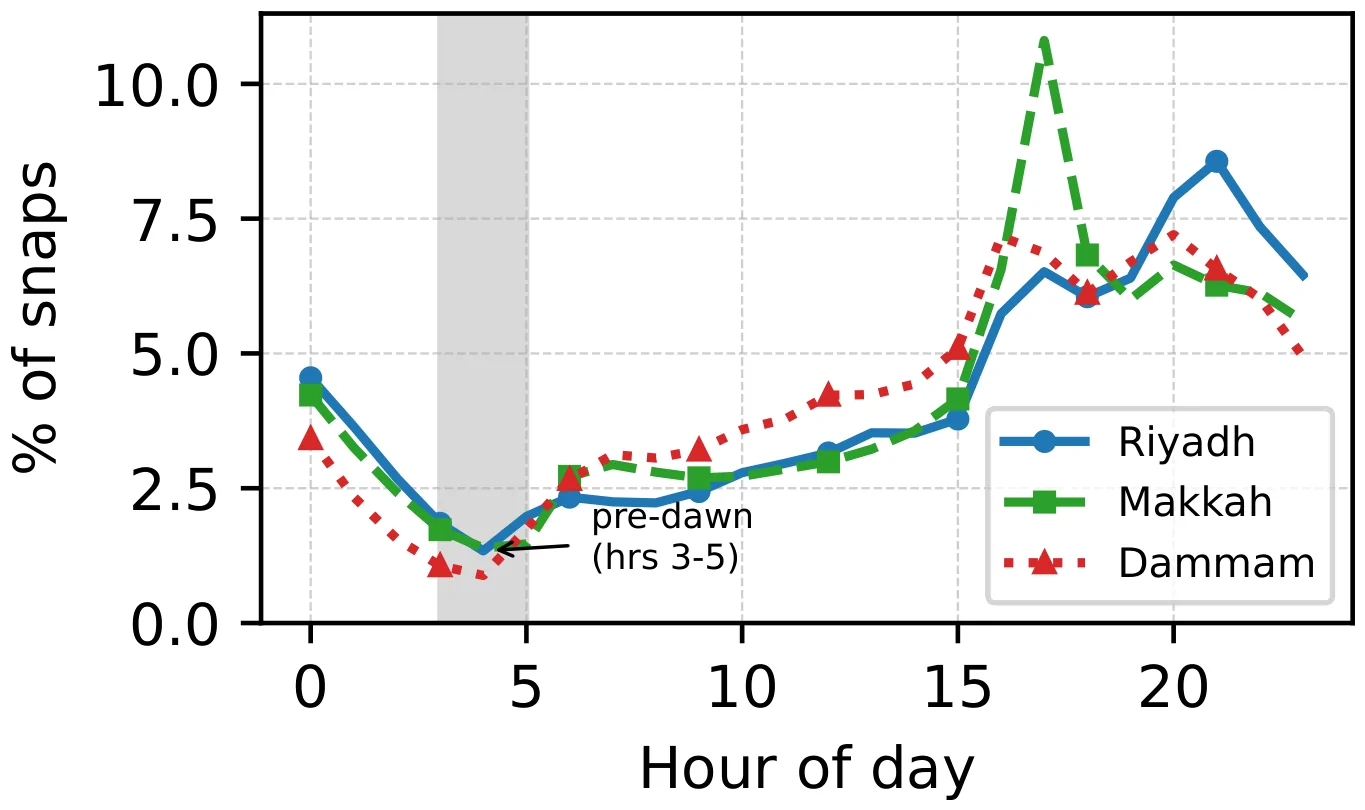
Hour-of-day activity (% of each city’s snaps, local time). Makkah peaks sharply at 17:00 (its busiest hour holds 10.8% of the day); Riyadh peaks in the evening; Dammam spans the working day. The pre-dawn hours 3–5 (shaded) are similar across all three cities (4–5%), so the separating feature is the afternoon/evening peak.

**Hour-of-day.** The diurnal profiles (Figure 4) separate the three cities. *Makkah* peaks sharply in the late afternoon: hour 17 alone accounts for 10.8% of its daily snaps, the most concentrated single-hour peak of the three cities. *Riyadh* peaks in the evening (hour 21, 8.6%). *Dammam* peaks across the working day and into the early evening (hour 20, 7.2%, atop a broad daytime plateau) and so has the *least* concentrated peak of the three. The pre-dawn hours are far more similar across cities than the afternoon and evening peaks. In hours 3–5, all three cities sit in a narrow band (Riyadh 5.2%, Makkah 4.6%, Dammam 3.7% of their respective daily snaps), so the diurnal contrast that separates the cities is the afternoon and evening peaks, not the pre-dawn floor.

**Figure 5:**
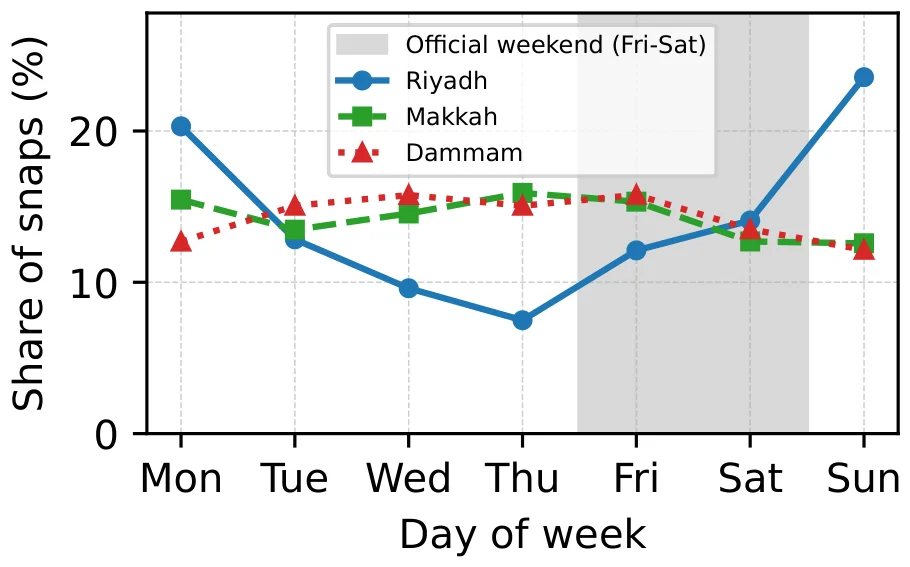
Day-of-week activity, normalized per weekday occurrence (% of each city’s snaps). The Saudi weekend (Friday–Saturday) is shaded.

**Day-of-week.** The weekday profiles (Figure 5, Table 5) are per-occurrence shares: each weekday’s value is the mean count over its occurrences in the window, renormalized to 100% (Section 4). The normalization matters. Raw weekday shares of these windows would count five Thursdays through Sundays against four Mondays through Wednesdays in Makkah and Dammam, inflating the later week by a quarter; on raw shares both cities appear to surge on Thursday–Friday, and this apparent surge largely dissolves under per-occurrence normalization. What remains is modest (Table 5): *Makkah* retains a mild Thursday lead and is lowest on Saturday and Sunday, and *Dammam* is flatter still—a working-week plateau with no Thursday–Friday distinction. *Riyadh*’s profile is dominated by its window’s composition rather than by a recurring rhythm: its 22 retained days contain only two Sundays and three Mondays, and the National Day period (the holiday Monday, 23 September, carries 12.3% of the window’s snaps, and its eve falls on one of the two observed Sundays) lifts Sunday to 23.6% and Monday to 20.3%. We accordingly read no recurring weekly rhythm from Riyadh’s window. Its weekday values also coincide with its collection history: daily volume rises twice in its window, after the collection began and again after its documented restart on 29 September [53], and each weekday occurs at most four times (Supplementary Table S4 gives the profile without National Day and its eve). The cross-city weekday comparison therefore rests on Makkah and Dammam. Overall, the day-of-week axis separates the cities far less than the raw shares suggested; the hour-of-day profile is the stronger temporal separator. These profiles describe autumn 2024. Ramadan, the Hajj season, and the summer holidays reorganize the Saudi week and its daily schedule, and they are the natural next windows for comparison.

### 5.3. Sensitivity Analyses

The comparisons above rest on three design choices that a reader should be able to probe: the windows are not matched in calendar time, one window contains a national holiday, and the concentration statistics run over each city’s own set of active tiles. This subsection varies each choice in turn. All values are computed the same way as the main results.

**Matched window.** The three collections overlap on exactly seven days, 3–9 October 2024, a span that contains each day of the week once and no holiday. Restricted to that shared week, the corpus still holds 212,406 Riyadh, 35,563 Makkah, and 49,242 Dammam snaps, and the Gini values become Riyadh 0.59, Makkah 0.56, and Dammam 0.44: Makkah no longer leads. The shared week is not a neutral sample: it opens the Makkah and Dammam collections, with means of 5080 and 7035 snaps/day against 8689 and 12,222 over their full windows, and closes Riyadh’s. Makkah’s Gini climbs as its window accumulates, with only brief dips along the way: 0.56 over the first seven days, 0.66 over fourteen, 0.69 over twenty-one, and 0.71 over the full thirty-two. The rise persists once collection start-up is excluded, from 0.57 to 0.72 (Section 5.4). Single-week snapshots of an ephemeral stream are unstable, and cross-city concentration comparisons should not be made from windows this short; the accumulation curve, not the seven-day value, is the informative object. Makkah’s lead in Table 4 is a property of sustained observation, in which the Grand Mosque core accumulates activity faster than the rest of the city.

**Matched observation length.** The matched week above equalizes *calendar* time, which Section 5.4 shows is far too short to be informative. The quantity that governs a concentration estimate is accumulated observation, and on that, the three cities are not comparable as collected: Riyadh is observed for 22 days and the other two for 32. Truncating every city to Riyadh’s 22 days—the shortest window in the study, so no city is extrapolated—gives Table 6. Makkah and Dammam both fall, by 0.010 and 0.012, while Riyadh is unchanged by construction, and the consequence falls on the Riyadh–Dammam comparison. At matched length the Riyadh–Dammam difference widens from 0.027 to 0.039; its 95% interval is [0.009, 0.068] under the tile bootstrap but [−0.035, 0.112] with 5 km blocks, so it is not resolved once spatial dependence is allowed for. Makkah’s lead over Riyadh, 0.107 at matched length, keeps intervals of [0.079, 0.133] and [0.043, 0.168].

**Holiday exclusion.** Removing National Day (23 September) from Riyadh’s window leaves its concentration metrics nearly unchanged: the Gini moves from 0.59 to 0.58, the top-1% share is unchanged at 6.8%, and the busiest tile drops from 2087 to 1855 snaps. The holiday’s leverage is temporal, not spatial: it dominates Riyadh’s weekly profile (Section 5.2) but not its concentration, which supports reading the holiday as a day-of-week confound rather than a distortion of the spatial results.

**Tile support.** The Gini over active tiles is conditional on how many tiles are active, and the identity $G^{\prime }=1-\frac{n}{n+Z}\left(1-G\right)$ makes the dependence explicit: adding *Z* zero-count tiles to a support of *n* active tiles raises the coefficient mechanically. Equalizing Dammam’s Gini with Riyadh’s would take only ∼55 additional zero-count tiles, 6.5% of Dammam’s support, while equalizing Riyadh’s with Makkah’s would take ∼884, a 39.8% enlargement. Makkah’s lead is therefore robust to any plausible support perturbation, and the Riyadh–Dammam difference is not.

**Spatial resolution.** Gini and top-share are computed over areal units and are therefore scale-dependent, the modifiable areal unit problem. The ∼1 km lattice is a collection parameter chosen for coverage and request limits rather than an analytical choice, so the ordering should not depend on it. Refinement below this spacing is not possible by design, because a snap’s position is known only to the query point that returned it, but coarsening is. Re-binning the raw coordinates onto ∼2 km and ∼3 km grids, with each city about its own origin and in metric rather than angular units, gives Table 7. Two things hold across all three resolutions. The ordering of the point estimates does not change, and Makkah stays well clear of the other two, its Gini exceeding Riyadh’s by 0.126 at 1 km, 0.120 at 2 km and 0.076 at 3 km. Coarsening raises every city’s coefficient, as expected when cells are merged, so the absolute values are resolution-dependent even though the comparison is not. The Riyadh–Dammam gap remains small at every resolution (0.018, 0.020, 0.037), and its sign is stable across scales. The re-aggregation reproduces the reported 1 km coefficients for Makkah (0.706) and Dammam (0.562), whose query points form a single 1 km lattice, but returns 0.580 for Riyadh against 0.589. Riyadh’s query set also holds 258 positions closer than 1 km to a neighbor, carrying 3.9% of its snaps, and 99 query-point labels that occur at two positions (709 snaps, 0.14%), so the assignment of snaps to tiles matters there. Keying on query-point labels, as in the main results, gives 2219 tiles and 0.589; keying on distinct stored coordinates gives 2274 and 0.599; and re-binning onto a fresh 1 km grid gives 2151 and 0.580. All three lie between Dammam and Makkah.

### 5.4. Measurement Requirements of the Sensor

The analyses so far describe what this sensor reports. Two further properties govern when those reports can be trusted, and both matter operationally because an ephemeral stream cannot be re-queried: a deployment that observed too briefly, or captured too little, cannot repair the deficit afterwards. We characterize each by re-analyzing the same corpus, so no additional collection is involved.

**Figure 6:**
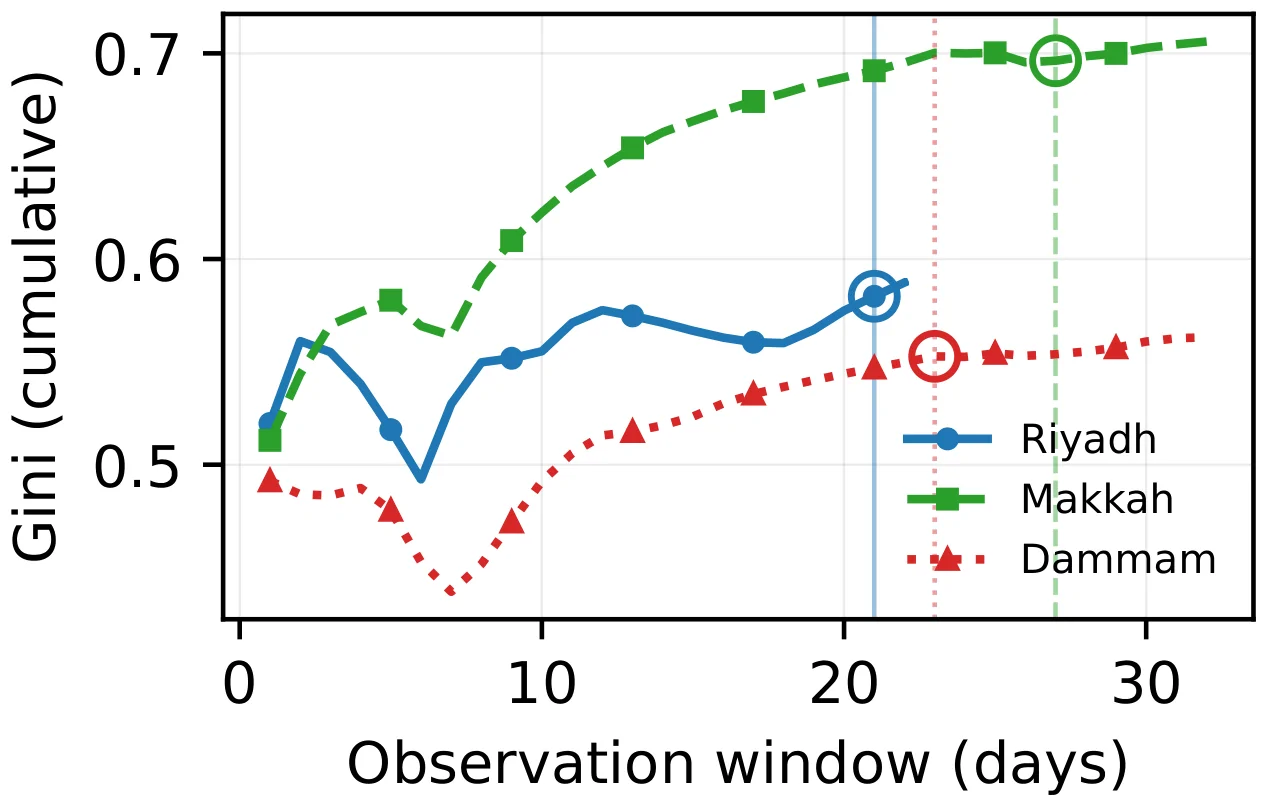
Integration time. Cumulative Gini as the observation window lengthens, with each city’s settle point circled and marked by a vertical rule. Line style and marker distinguish the three cities, as given in the legend. All three stay below their final value throughout, dipping before they climb; Riyadh’s 22-day window barely reaches its own.

**Integration time.** Recomputing the concentration statistics over the corpus accumulated from the first day of a window through day *k*, for every *k*, traces how the estimate approaches its final value (Figure 6). The approach is slow, and in no city does the running estimate ever exceed its final value—but nor is it a steady climb: all three fall before they rise, Riyadh to 0.493 by day 6 and Dammam to 0.438 by day 7, each below its own first-day value. End to end, Riyadh’s Gini goes from 0.52 to 0.589, Makkah’s from 0.51 to 0.706, and Dammam’s from 0.49 to 0.562. Taking the *integration time* to be the smallest *k* beyond which the running estimate never again departs from its final value by more than 0.01, Dammam settles after 23 days of its 32 and Makkah after 27 of its 32. Riyadh’s series reaches the same criterion on day 21 of 22, but that value is right-censored rather than measured: it is tested against a single later observation, and Riyadh is still climbing when its window closes, gaining 0.0067 on its final day—two thirds of the whole tolerance—against 0.0014 for Makkah and 0.0006 for Dammam. Riyadh’s integration time is therefore a lower bound, and its concentration estimate is the least converged of the three. The settle point depends on the tolerance: at 0.02, Dammam and Makkah settle after 20 days, while at 0.005, both reach the band only on day 30 of 32, too late to confirm (Supplementary Table S2). Part of the settling time is collection start-up. Restarted once its daily active-tile count had stabilized (7 October for Dammam, 8 October for Makkah), each curve settles at 0.01 after 12 and 14 days, respectively (24 at 0.005; 6 and 10 at 0.02); Riyadh’s restarted series is too short to evaluate.

The requirement is not uniform across the three cities, and the matched week above marks its limit. Riyadh’s seven-day slice of 3–9 October yields 0.585 against its full-window 0.589, a gap of 0.004 that sits inside both the settling tolerance and the bootstrap standard error, whereas the same seven days give 0.563 for Makkah against 0.706 and 0.438 for Dammam against 0.562. A week suffices for Riyadh and falls far short for the other two. What separates them is the state of the deployment: Riyadh’s own first seven days, when its grid was still activating, give 0.530, and its active-tile count climbs from 629 to 1943 over the first six days, reaching 95% of its final support only on day 14. The early part of a cumulative curve therefore records the collection warming up as much as the stream integrating, which is also why all three cities dip before they rise. Read together, the two analyses suggest that what governs the deficit is how much activity has accumulated rather than how many days have passed: once a deployment’s active support stops growing, a single week of a high-volume city reproduced its long-run concentration here, while the lower-volume cities needed about two more weeks for the busiest tiles to separate. Three campaigns in three cities cannot establish a general threshold, and 23 to 27 days is the range confirmed here—in the two cities observed long enough to confirm it—rather than a requirement for Snap Map deployments at large. This also explains the matched-window reversal above.

**Figure 7:**
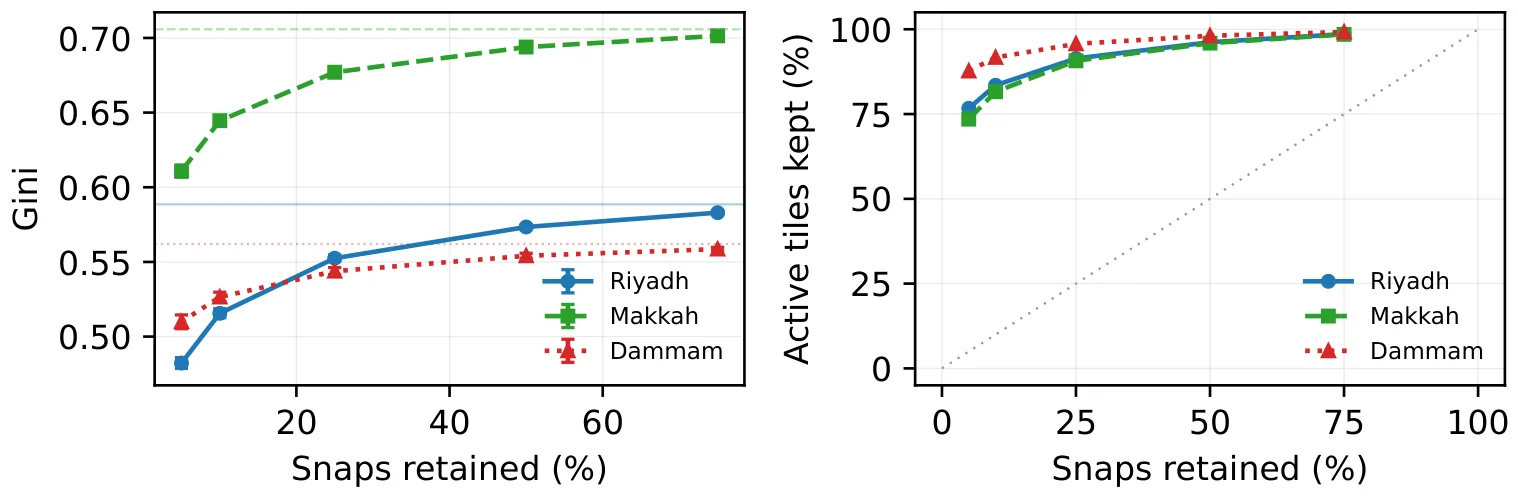
Sampling sufficiency under binomial thinning. (**Left**): Gini against retention rate; error bars are one standard deviation over 200 repetitions, and horizontal rules mark the full-corpus values. (**Right**): Share of active tiles retained, with the diagonal marking proportional loss; all three cities lie far above it.

**Sampling sufficiency.** The corpus records what the platform made public, which is an unknown fraction of what was posted, and no ground truth is available to fix that fraction. Its consequences can nevertheless be bounded by simulating it: retaining each snap independently with probability *p* models a partially visible stream in which visibility is independent across snaps. Repeating this 200 times per rate (Table 8, Figure 7) shows that the Gini is biased *downward* under partial capture, decreasing by −0.11 for Riyadh at 5% retention but only −0.015 at 50%. The direction is expected from theory, the Gini being downward-biased in small samples [57]; what the table adds is its magnitude and its source. Active-tile counts degrade far more gracefully than proportionally—5% of Riyadh’s snaps still touch 76.7% of its tiles, because the sparse tail is wide and shallow, and 73.5% of Makkah’s and 87.7% of Dammam’s remain occupied—so the bias accrues from thinning counts within surviving tiles rather than from losing them. The bias is smallest for Dammam at every rate (−0.052 at 5% retention, against −0.106 for Riyadh and −0.095 for Makkah), consistent with its snaps sitting in relatively few busy tiles whose counts stay large after thinning, and the Riyadh and Dammam curves cross between 25% and 10% retention (0.552 against 0.544 at 25%; 0.516 against 0.526 at 10%). It is also informative, within the assumption the simulation makes: if the platform surfaces an approximately item-independent subset of activity, the true concentration is higher than what is reported here, so the estimates in Table 4 are biased downward and therefore conservative. Clustered loss behaves differently (Supplementary Table S3): when whole tile-days are dropped, the Gini of Riyadh and Dammam rises slightly at low retention (+0.008 and +0.021 at 5%) while Makkah’s falls (−0.059), so the direction of the bias depends on how visibility is lost. Most usefully, the cross-city comparison degrades unevenly. Makkah’s lead is intact at every rate tested under both schemes, surviving the loss of 95% of the corpus, whereas the Riyadh–Dammam ordering inverts at and below 10% retention under item-independent loss and narrows to 0.014 at 5% under clustered loss. Partial platform visibility is therefore capable of overturning the second comparison but not the first, which is the same boundary the bootstrap intervals and the support margins draw, reached here by an independent route.

## 6. Discussion

The three cities differ in measurable ways. Makkah’s concentration lead on every spatial measure, its extreme Grand Mosque tile, and its sharp late-afternoon peak describe activity gathered around a single core. The late-afternoon concentration is compatible with this city’s distinctive activity schedule, but Snap Map timing alone cannot separate a visitation pattern from the ordinary late-afternoon rise in posting. Riyadh and Dammam differ in *how* rather than *how much* they concentrate: Dammam gathers activity into a strong head of busy tiles with the highest median, while Riyadh spreads a comparable total across nearly three times as many active tiles with an evening peak. On the day-of-week axis the separation is modest once weekday occurrence is normalized for, and we read no strong weekly story from it. The U.S. Snap Map study of Juhász and Hochmair [21] reported strong concentration across three broadly similar metros, with a dominant core in each and secondary clusters in two of the three. On the one scalar the two studies share, their pooled top-1% figure of about a quarter stands well above every value measured here (Riyadh 6.8%, Dammam 7.3%, Makkah 10.7%). This gap is not evidence that Saudi cities concentrate less: their tiles are roughly 2.4 km across against the ∼1 km used here, and a coarser tile absorbs more of a city into each cell, so the comparison is one of spatial support as much as of behavior. Our results extend the measurement to a Gulf setting and qualify the picture: the degree of concentration is not universal, ranging here from a Gini of 0.71 [0.68, 0.73] in Makkah to values near 0.56–0.59 for the other two cities, whose difference from each other is within sampling uncertainty. The Gulf Snap Map study closest to ours examined the Grand Mosque alone [13]. Placing that site inside a cross-city concentration frame shows that its extreme density is not a platform-wide norm: on every measure it stands far above the busiest locations of the two other Saudi metros measured under the same methodology. The weekday analysis also carries a methodological caution for the temporal-rhythm literature, which tends to assume a Saturday–Sunday weekend and whole-week windows [25]. In windows that are not multiples of seven days, raw weekday shares manufacture spurious peaks; here, an apparent Thursday–Friday surge dissolves under per-occurrence normalization. The local Friday–Saturday weekend also means that imported weekly templates misalign by construction. VGI activity signatures must therefore be read in the context of a city’s morphology, calendar, and observation window rather than assumed uniform. In practical terms, these measurements offer a high-frequency, place-resolved *complement* to surveys and footfall counters, subject to the platform-visibility and demographic caveats of Section 7.

The week that all three windows share shows the requirement at work. Over 3–9 October, Makkah’s Gini is 0.56, below Riyadh’s 0.59; Makkah’s own estimate rises from 0.56 over its first seven days to 0.71 over the full window. That week opens the Makkah and Dammam collections and closes Riyadh’s. Read as an instrument, the stream has a usable but narrow operating envelope, and Section 5.4 puts numbers to it for these campaigns. In the two campaigns long enough to confirm it, estimates stabilized after 23 to 27 days at a tolerance of 0.01 (20 days at 0.02), or 12 to 14 days once collection start-up had passed. Shorter windows gave lower values in all three cities, a deficit that cannot be recovered afterwards from an ephemeral source. Robustness to incomplete capture then depends on what is being asked. A large concentration gap is recoverable from a small fraction of the stream, so studies asking whether a city has a dominant core are on firm ground even under heavy loss; a narrow gap between two cities is not, and requires near-complete capture. That the thinning experiment, the bootstrap intervals, and the support margins independently converge on the same dividing line—Makkah separable, Riyadh and Dammam not—indicates that this distinction is robust across the analytical choices examined in these campaigns. These bounds are derived on Snap Map.

## 7. Limitations and Ethics

**Sampling.** The platform tile service is undocumented and may return a subset of posted snaps, so the corpus is a lower bound on public activity and the absolute sampling fraction against ground truth is unknown. The repeat-query analysis (Section 3.5) shows that coverage is *stable* in all three grids: 96.9% and 96.5% of the items returned in Makkah and Dammam and 94.8% of the ≈4.8 M item observations of the Riyadh probe were duplicates, so polling an already-populated grid saturates rather than endlessly surfacing hidden snaps. Stability is not the same as completeness, so we describe the study as a measurement of *platform-visible* activity rather than of all activity. Any residual sampling bias that is shared across cities, such as a constant exposure rate, vanishes in the within-city normalized comparisons that carry our main temporal claims.

**Unequal windows and the holiday confound.** Collection windows differ across cities (Riyadh 22 days vs. Makkah and Dammam 32 days), and Riyadh’s shorter window contains Saudi National Day. The windows were not simultaneous because the collection platform was developed and validated on Riyadh first and then extended to Makkah and Dammam, so city is confounded with season, calendar, and collection stage: on any shared date the collections were at different points of their start-up. We address this in three ways. (i) All cross-city *temporal* comparisons use within-city normalized shares (Section 4), which are first-order insensitive to window length. (ii) For the volume comparisons that remain (busiest-tile counts) we report per-day rates alongside the raw counts. Makkah’s core leads on both the raw count (3.5× Riyadh) and the per-day rate (2.4×), so this conclusion does not depend on window length. (iii) For the concentration metrics, the Gini coefficient and top-1% share are computed over the *spatial* distribution of per-tile totals, and they are invariant to window length only under a spatially uniform change: they shift whenever a longer window changes the *shape* of the spatial distribution. That condition does not hold here, and we do not rely on it: the integration analysis in Section 5.4 shows the running Gini drifting by 5.6 to 17.8 bootstrap standard errors as a window lengthens, because the busiest tiles accumulate faster than the rest. Accumulation in this corpus is not spatially uniform, so scale invariance licenses nothing, and the direction of the resulting bias is knowable rather than merely uncertain: estimates approach their final value from below and Riyadh has the shortest window, so Riyadh’s published figure is the most understated of the three. Rather than argue that window length does not matter, we equalize it and report the comparison at matched observation length. The holiday mainly perturbs Riyadh’s day-of-week profile, which we flag as a confound rather than interpret, and a single-day event spread over Riyadh’s many active tiles has limited leverage on its city-wide Gini (0.59). Neither sensitivity comparison removes the confound of calendar and collection stage; because the stream cannot be recovered retrospectively, matched dates require simultaneous deployment, which we recommend for future collections.

**Other limitations.** Ephemerality means that deleted or expired snaps are absent from the public record at collection time. The measured population is a single platform’s users, skewed toward the demographics that adopt Snapchat and choose to post publicly to the map; the measurements characterize *platform-visible* activity, and any inference to the activity of city populations at large inherits that selection, which the data cannot correct. The three cities, while functionally contrasting, are all Saudi, and with one city per urban role, the associations reported here cannot separate a role effect from any other between-city difference. Integration times and thinning results describe these campaigns under the stated assumptions, and the Riyadh–Dammam comparison also depends on the tile definition (Section 5.3). The analysis treats each snap as a geotagged, timestamped event and does not use the image or video content itself, so the activity it reads is positional and temporal rather than semantic.

**Ethics.** This study analyzes publicly accessible Snap Map metadata and uses no image, video, textual, or audio content. The collected metadata carry no user account identifiers, and the study involves no interaction with, or profiling of, individuals. All analyses are performed at the aggregate place and time level, and only aggregate statistics are reported. Raw Snap Map media are not redistributed.

## 8. Conclusions

This article characterized public Snap Map activity as an opportunistic urban sensor, across an administrative, a religious, and an industrial and coastal Saudi city, using a corpus of more than 1.18 million public snaps. It compared Snap Map spatial concentration across cities as a scalar with stated uncertainty under one methodology and reported what such a measurement required of these deployments. The results are divided into what the sensor reports and what it needs to report it. On the first, Makkah is the most spatially concentrated city on every measure (Gini 0.71 [0.68, 0.73]), with a single dominant core at the Grand Mosque, and its lead is robust to tile and spatial-block resampling, tile-support perturbation, holiday exclusion, and the loss of 95% of the corpus. Riyadh and Dammam are close in degree and concentrate in different shapes: Dammam in a strong head of busy tiles and Riyadh in a long active tail. Hour-of-day profiles separate the three cities cleanly, while occurrence-normalized day-of-week profiles separate them only weakly, and we showed that raw weekday shares in non-whole-week windows manufacture spurious surges. On the second, concentration estimates took 23 to 27 days to stabilize in the two campaigns observed long enough to confirm it and 12 to 14 days once collection start-up had passed, approaching their final value from below, so short deployments understated concentration, and under approximately item-independent partial visibility the reported Gini values are biased downward and therefore conservative. The analyses behind these requirements form a count-based procedure (Section 4.3). A natural next step is to classify the visual content of each snap with a vision–language model [58,59] and test whether scene categories line up with the spatial and temporal signatures measured here, extending the grid to additional Gulf cities and to Ramadan and Hajj calendars whose rhythms differ from the ordinary weeks sampled in this study.

## Figures and Tables

**Table 1:** Positioning relative to comparable volunteered geographic information (VGI) and Snap Map studies.

Study	Source	Region	Conc. Metric
Crandall et al. [6]	Flickr	Global	no
Noulas et al. [7]	Foursquare	Multi	no
Cranshaw et al. [8]	Check-ins	1 city	no
Frias-Martinez [24]	Twitter	City-scale	no
Juhász & Hochmair [21]	Snap Map	US (3)	top-1%
Alghamdi et al. [13]	Snap Map	Makkah (1 site)	no
**This work**	Snap Map	Gulf (3)	Gini + CI

**Table 2:** Three-city Snap Map datasets (2024), from catalog to analyzed corpus.

	Riyadh (Admin.)	Makkah (Religious)	Dammam (Ind./Coastal)
Catalog records	534,389	280,803	393,910
In core window	511,918	278,044	391,103
Low-coverage days removed	1 (480 snaps)	0	0
Duplicates removed	0	0	0
Snaps analyzed	511,438	278,044	391,103
Collection days	22	32	32
Active tiles	2219	1049	848
Median snaps/tile	152.0	67.0	298.0
Max snaps/tile	2087	**7379**	5573
Video share (%)	80.4	76.4	74.4

Bold marks the single busiest tile across the three cities.

**Table 3:** Fields of a catalog record used in this study, with synthetic example values.

Field	Content	Synthetic Example
id	Platform media identifier (deduplication key)	EXAMPLE0001
location_id	Label of the first-retrieving query point	Riyadh_0001
latitude/longitude	Coordinates of that query point	24.7000, 46.7000
timestamp	Server time, epoch milliseconds (UTC)	1727000000000
snapMediaType	Image or video	VIDEO
neighborhood	District containing the query point	Example Dist.

**Table 4:** Spatial-concentration metrics. Brackets are 95% CIs from a nonparametric bootstrap over tiles and over 5 km spatial blocks (5000 resamples each). The busiest tile’s per-day rate (max tile ÷ retained collection days) is given in the text.

City	Gini	CI, Tiles	CI, Blocks	Top-1% [CI, Tiles]	Median/Max Tile
Makkah	**0.71**	[0.68, 0.73]	[0.66, 0.74]	10.7% [7.7, 14.6]	67.0/7379
Riyadh	0.59	[0.57, 0.60]	[0.54, 0.63]	6.8% [6.1, 7.4]	152.0/2087
Dammam	0.56	[0.54, 0.59]	[0.50, 0.62]	7.3% [6.0, 8.8]	298.0/5573

Bold marks the highest Gini coefficient in the column.

**Table 5:** Day-of-week activity, normalized per weekday occurrence (% of each city’s snaps; maximum in bold). Occurrences: Makkah and Dammam, five Thu–Sun, four Mon–Wed; Riyadh, Mon 3, Tue 4, Wed 4, Thu 3, Fri 3, Sat 3, Sun 2.

Day	Riyadh	Makkah	Dammam
Mon	20.3	15.5	12.7
Tue	12.8	13.5	15.1
Wed	9.6	14.5	**15.8**
Thu	7.5	**15.9**	15.1
Fri	12.1	15.3	**15.8**
Sat	14.1	12.7	13.5
Sun	**23.6**	12.6	12.1

Bold marks the highest share in each row.

**Table 6:** Concentration at matched observation length (every city truncated to 22 days), with 95% CIs for each city (tiles) and for its difference from Riyadh (tiles; 5 km blocks).

City	Days	Tiles	Gini [CI, Tiles]	Diff. [CI, Tiles]	Diff. CI, Blocks
*As collected*					
Riyadh	22	2219	0.589 [0.574, 0.603]	—	—
Makkah	32	1049	0.706 [0.684, 0.727]	+0.117 [0.092, 0.143]	[0.053, 0.177]
Dammam	32	848	0.562 [0.537, 0.585]	−0.027 [−0.054, 0.003]	[−0.100, 0.047]
*Matched at 22 days of accumulation*					
Riyadh	22	2219	0.589 [0.574, 0.603]	—	—
Makkah	22	988	0.696 [0.673, 0.718]	+0.107 [0.079, 0.133]	[0.043, 0.168]
Dammam	22	834	0.550 [0.525, 0.575]	−0.039 [−0.068, −0.009]	[−0.112, 0.035]

Italics name the two window definitions compared. An em dash denotes a quantity that is not defined for that row.

**Table 7:** Concentration at three tile sizes, re-aggregated from raw coordinates over each city’s core window. Coarsening raises every coefficient, but the ordering of the point estimates is unchanged and Makkah’s separation persists.

City	Cell	Active Cells	Gini	Top 1% (%)
Riyadh	∼1 km	2151	0.580	6.8
Riyadh	∼2 km	657	0.610	6.1
Riyadh	∼3 km	329	0.641	5.1
Makkah	∼1 km	1049	**0.706**	10.7
Makkah	∼2 km	350	**0.730**	10.9
Makkah	∼3 km	178	**0.717**	9.0
Dammam	∼1 km	848	0.562	7.3
Dammam	∼2 km	299	0.590	5.6
Dammam	∼3 km	156	0.604	6.5

Bold marks the highest Gini coefficient for each city across cell sizes.

**Table 8:** Sampling sufficiency under binomial thinning (200 repetitions per rate, seed 1464). Gini bias is relative to the full-corpus value; the **Tiles** columns give the share of active tiles still occupied after thinning, except in the *Full* row, which gives the underlying active-tile counts.

Retained	Riyadh		Makkah		Dammam	
	Gini (Bias)	Tiles	Gini (Bias)	Tiles	Gini (Bias)	Tiles
5%	0.482 (−0.106)	76.7%	0.611 (−0.095)	73.5%	0.510 (−0.052)	87.7%
10%	0.516 (−0.073)	83.5%	0.645 (−0.061)	81.6%	0.526 (−0.035)	91.7%
25%	0.552 (−0.036)	91.4%	0.677 (−0.029)	90.7%	0.544 (−0.018)	95.7%
50%	0.573 (−0.015)	96.2%	0.694 (−0.012)	95.9%	0.554 (−0.008)	98.1%
75%	0.583 (−0.006)	98.6%	0.701 (−0.004)	98.5%	0.559 (−0.003)	99.2%
Full	0.589	2219	0.706	1049	0.562	848

## Data Availability

The hourly tile-level snap counts for Riyadh, Makkah, and Dammam that support the findings, with surrogate tile identifiers, and a notebook that computes every reported number are archived on Zenodo [56] under a CC BY 4.0 license. Tile coordinates, per-snap records, and Snap Map media are not released because of privacy and platform-related considerations. The collection platform is described in the companion paper [53], and its Riyadh aggregate record is available at [54]. District-boundary polygons used for collection are publicly available at [55].

## Supplementary Materials

The following supporting information can be downloaded at https://www.mdpi.com/article/10.3390/s26217018/s1. Table S1: Gini coefficients and between-city differences with 95% bootstrap intervals over tiles and over 5 and 10 km spatial blocks; Table S2: Integration time at three tolerances, from the first day of each window and on curves restarted after collection start-up; Table S3: Clustered thinning of whole tile-days; Table S4: Riyadh day-of-week profile with and without Saudi National Day and its eve.
