# Supplementary material for: Public Snap Map Activity as an Opportunistic Urban Sensor: Spatial Concentration, Temporal Rhythms, and Measurement Requirements Across Three Saudi Cities
Source: Sensors (Basel). 2026 Nov 6;26(21):7018. doi: 10.3390/s26217018 (PMC13766423; doi:10.3390/s26217018)
Supplement: Supplementary file 1 [file sensors-26-07018-s001.zip › sensors-4567163-supplementary.pdf]

# Supplementary Materials

## Public Snap Map Activity as an Opportunistic Urban Sensor: Spatial Concentration, Temporal Rhythms, and Measurement Requirements across Three Saudi Cities

Mohammed Almukaynizi and Abdulaziz Alajaji

Every value below is produced by the archived analysis code from the same tile-day tables as the main text.

**Table S1:** Gini coefficients and between-city differences with 95% bootstrap intervals over tiles and over square spatial blocks of 5 and 10 km (5,000 resamples, seed 1464). Blocks per city at 5 and 10 km: Riyadh 142 and 45; Makkah 79 and 25; Dammam 74 and 25.

| | **Estimate** | **Tiles** | **5 km Blocks** | **10 km Blocks** |
|---|---|---|---|---|
| *As collected* | | | | |
| Riyadh | 0.589 | [0.574, 0.603] | [0.542, 0.633] | [0.514, 0.663] |
| Makkah | 0.706 | [0.684, 0.727] | [0.661, 0.744] | [0.648, 0.747] |
| Dammam | 0.562 | [0.537, 0.585] | [0.505, 0.619] | [0.481, 0.647] |
| Makkah−Riyadh | 0.117 | [0.092, 0.143] | [0.053, 0.177] | [0.022, 0.202] |
| Riyadh−Dammam | 0.027 | [−0.003, 0.054] | [−0.047, 0.100] | [−0.085, 0.143] |
| Makkah−Dammam | 0.144 | [0.111, 0.177] | [0.073, 0.211] | [0.040, 0.232] |
| *Matched at 22 days* | | | | |
| Riyadh | 0.589 | [0.574, 0.603] | [0.541, 0.635] | [0.510, 0.662] |
| Makkah | 0.696 | [0.673, 0.718] | [0.652, 0.735] | [0.640, 0.738] |
| Dammam | 0.550 | [0.525, 0.575] | [0.494, 0.605] | [0.469, 0.636] |
| Makkah−Riyadh | 0.107 | [0.079, 0.133] | [0.043, 0.168] | [0.014, 0.192] |
| Riyadh−Dammam | 0.039 | [0.009, 0.068] | [−0.035, 0.112] | [−0.074, 0.147] |
| Makkah−Dammam | 0.146 | [0.112, 0.180] | [0.076, 0.215] | [0.045, 0.236] |

**Table S2:** Integration time (days to settle) at three tolerances, from the first day of each window and on curves restarted after collection start-up (the first day opening seven consecutive days with at least 85% of the final-14-day median of daily active tiles) or after dropping the first seven days. [c]Settle point within the last two days of the series, right-censored.

| City | Curve | Start | Days | 0.005 | 0.01 | 0.02 |
|---|---|---|---|---|---|---|
| Riyadh | From day 1 | 2024-09-17 | 22 | 22[c] | 21[c] | 20[c] |
| | After start-up | 2024-10-03 | 7 | 7[c] | 7[c] | 6[c] |
| | First 7 days dropped | 2024-09-24 | 15 | 15[c] | 14[c] | 13[c] |
| Makkah | From day 1 | 2024-10-03 | 32 | 30[c] | 27 | 20 |
| | After start-up | 2024-10-08 | 27 | 24 | 14 | 10 |
| | First 7 days dropped | 2024-10-10 | 25 | 23[c] | 12 | 9 |
| Dammam | From day 1 | 2024-10-03 | 32 | 30[c] | 23 | 20 |
| | After start-up | 2024-10-07 | 28 | 24 | 12 | 6 |
| | First 7 days dropped | 2024-10-10 | 25 | 7 | 6 | 1 |

**Table S3:** Clustered thinning: whole tile-days kept with probability $p$ (200 repetitions per rate, seed 1464). Mean Gini (bias relative to the full corpus) and share of active tiles still occupied.

| | Riyadh | | Makkah | | Dammam | |
|---|---|---|---|---|---|---|
| Retained | Gini (bias) | Tiles | Gini (bias) | Tiles | Gini (bias) | Tiles |
| 5% | 0.597 (+0.008) | 38.6% | 0.647 (−0.059) | 50.4% | 0.583 (+0.021) | 64.8% |
| 10% | 0.602 (+0.013) | 59.4% | 0.663 (−0.043) | 68.0% | 0.583 (+0.021) | 83.2% |
| 25% | 0.595 (+0.007) | 83.1% | 0.676 (−0.030) | 83.9% | 0.566 (+0.004) | 93.3% |
| 50% | 0.588 (−0.001) | 93.0% | 0.691 (−0.015) | 92.5% | 0.560 (−0.002) | 97.0% |
| 75% | 0.588 (−0.001) | 97.3% | 0.700 (−0.006) | 97.1% | 0.561 (−0.002) | 98.8% |
| Full | 0.589 | | 0.706 | | 0.562 | |

**Table S4:** Riyadh day-of-week profile (% per weekday occurrence) with and without Saudi National Day (23 September 2024) and its eve (22 September), with the number of occurrences of each weekday.

| | All 22 days | | Without 22–23 Sep | |
|---|---|---|---|---|
| Day | % | n | % | n |
| Mon | 20.3 | 3 | 13.5 | 2 |
| Tue | 12.8 | 4 | 14.4 | 4 |
| Wed | 9.6 | 4 | 10.8 | 4 |
| Thu | 7.5 | 3 | 8.4 | 3 |
| Fri | 12.1 | 3 | 13.6 | 3 |
| Sat | 14.1 | 3 | 15.8 | 3 |
| Sun | 23.6 | 2 | 23.4 | 1 |
